# Charged Amino Acids Contribute to ZorO Toxicity

**DOI:** 10.3390/toxins15010032

**Published:** 2022-12-31

**Authors:** Bikash Bogati, Selene F. H. Shore, Thomas D. Nipper, Oana Stoiculescu, Elizabeth M. Fozo

**Affiliations:** Department of Microbiology, University of Tennessee Knoxville, Knoxville, TN 37996, USA

**Keywords:** type I toxin-antitoxin, small protein, ZorO, charged residues, aminoglycosides

## Abstract

Chromosomally encoded toxin-antitoxin systems have been increasingly identified and characterized across bacterial species over the past two decades. Overproduction of the toxin gene results in cell growth stasis or death for the producing cell, but co-expression of its antitoxin can repress the toxic effects. For the subcategory of type I toxin-antitoxin systems, many of the described toxin genes encode a small, hydrophobic protein with several charged residues distributed across the sequence of the toxic protein. Though these charged residues are hypothesized to be critical for the toxic effects of the protein, they have not been studied broadly across different type I toxins. Herein, we mutated codons encoding charged residues in the type I toxin *zorO*, from the *zor-orz* toxin-antitoxin system, to determine their impacts on growth inhibition, membrane depolarization, ATP depletion, and the localization of this small protein. The non-toxic variants of ZorO accumulated both in the membrane and cytoplasm, indicating that membrane localization alone is not sufficient for its toxicity. While mutation of a charged residue could result in altered toxicity, this was dependent not only on the position of the amino acid within the protein but also on the residue to which it was converted, suggesting a complex role of charged residues in ZorO-mediated toxicity. A previous study indicated that additional copies of the *zor-orz* system improved growth in aminoglycosides: within, we note that this improved growth is independent of ZorO toxicity. By increasing the copy number of the *zorO* gene fused with a FLAG-tag, we were able to detect the protein expressed from its native promoter elements: an important step for future studies of toxin expression and function.

## 1. Introduction

First identified in plasmids, type I toxin-antitoxin (TA) systems are also found in bacterial chromosomes. The toxin gene encodes for a small protein (usually less than 60 amino acids) and the antitoxin gene encodes for a small RNA (sRNA). While overproduction of the toxin often results in growth inhibition and cell lysis, co-expression of the sRNA neutralizes the toxicity. This is achieved by sRNA complementary base pairing with the toxin-encoding mRNA and subsequent prevention of toxin translation and/or stimulation of degradation of the toxin mRNA [1,2]. Despite their small size, overproduction studies indicate that not all type I toxins impact cells via the same mechanism [1,2,3].

Some type I toxins have cellular targets such as RNA (SymE) and DNA (RalR), whereas several others have been experimentally validated to either localize to the bacterial inner membrane or have a predicted alpha-helical structure [3,4,5]. For the *hokB/sokB* and *tisB/istR1* TA systems, toxins HokB and TisB were shown to localize to the inner membrane of *Escherichia coli* and result in pore formation upon their overproduction [6,7,8]. The 49 amino acid HokB forms a dimer via disulfide interactions resulting in pore formation that dissipates membrane potential and leakage of ATP from the cell [6,9]. While very high levels of HokB can result in cell death, at lower production levels, cells are better able to survive lethal concentrations of the antibiotics tobramycin and ofloxacin: such cells are referred to as persister cells [10]. Overproduction of the 29 amino acid TisB toxin also permits ion conductance via the formation of a dimer of dimers giving rise to a tetrameric charged zipper structure [7,11]. This results in membrane depolarization, a reduction in cellular ATP levels, and subsequent persistence upon lethal doses of antibiotics [8,12,13,14]. Transcription of *tisB* occurs in response to DNA damage as it is normally repressed by the SOS response regulator (LexA); thus, it is speculated that TisB production during SOS is needed to allow the cell time to repair DNA damage before replicating its chromosome [8,15].

Apart from type I toxins, overproduction of de novo synthesized small hydrophobic proteins has been shown to cause membrane depolarization and affect aminoglycoside uptake resulting in an increase in the minimum inhibitory concentration (MIC) for those antibiotics in *E. coli* [16]. Despite their effect on membrane depolarization and cellular growth, neither the type I toxin IbsC nor LdrD resulted in an increased MIC against aminoglycosides [16,17,18]. However, our previous work showed that the type I TA locus, *zor-orz,* when cloned onto a medium copy plasmid, was able to improve the growth of *E. coli* in the presence of kanamycin and other aminoglycoside antibiotics as well as increase the MIC for kanamycin and gentamycin [19]. 

While many type I toxins are predicted to localize to the cytoplasmic membrane, there are variations in the predicted orientations within the membrane and the length of the predicted transmembrane domain [3]. Many membrane-associated type I toxins have a global positive charge [3], which can play an important role in associating to the negatively charged bacterial membrane and/or with interactions to other proteins. For some toxins, both positively and negatively charged amino acid residues are distributed within the toxin sequence. The interactions between such charged amino acids in TisB lead to the formation of oligomers that form small pores in the membrane [7]. On the contrary, other toxins will associate with the membrane upon overproduction, but their primary effects are likely not on the membrane. For example, the overproduction of membrane-localizing type I toxins Fst or BsrG result in either nucleic acid condensation or inhibition of cell wall synthesis [20,21,22]. Specific regions within a type I toxin protein, such as a conserved hydrophobic domain of the Fst toxin within enterococci, are highly sensitive to mutation rendering them non-toxic [23]. Likewise, the highly hydrophobic center of the IbsC toxin was the determinant for the toxicity of this protein [24]. Identification of critical residues within a toxin may aid in distinguishing between potential benefits from production versus possible artificial toxic effects from the overproduction of a small, hydrophobic protein. 

ZorO is the toxin component of a bacterial type I TA system, *zorO-orzO*, first identified in the chromosome of *Escherichia coli* O157:H7 EDL933 [25,26]. It is located in a region known as the *zor-orz* locus, which encodes, in tandem, a second highly homologous type I TA gene pair *zorP-orzP* [25,26]. ZorO translation is regulated by a sRNA, OrzO, via complementary base pairing with the *zorO* mRNA, and also by secondary structure formed within the long 5′ untranslated region (UTR) of its mRNA [19,25,27]. Upon translation, the ZorO protein localizes to the inner membrane [19,28]. Ectopic overexpression of *zorO* from plasmids results in growth stasis, membrane depolarization, and ATP depletion in *E. coli* MG1655 (which does not naturally encode *zorO*) without a significant impact on bacterial gross morphology [19]. Although toxic when overproduced, cells harboring multiple copies of the entire *zor-orz* locus (including the toxins, antitoxins and their native promoter elements) were able to grow significantly better in aminoglycosides [19].

We hypothesized that the charged amino acids in the ZorO sequence are essential for the effects observed after ZorO overproduction. To determine this, we overexpressed mutants of *zorO* and identified key amino acids that conferred toxicity. We found that changes in several charged amino acids either decreased or completely abolished ZorO toxicity despite localization to the membrane. However, a nontoxic variant could improve growth in kanamycin, suggesting that membrane depolarization is not needed for *zorO-orzO* induced protection from aminoglycosides. 

## 2. Results

### 2.1. Alterations of Charged Residues of ZorO Do Not Impact Predicted Hydrophobicity

**Figure 1:**
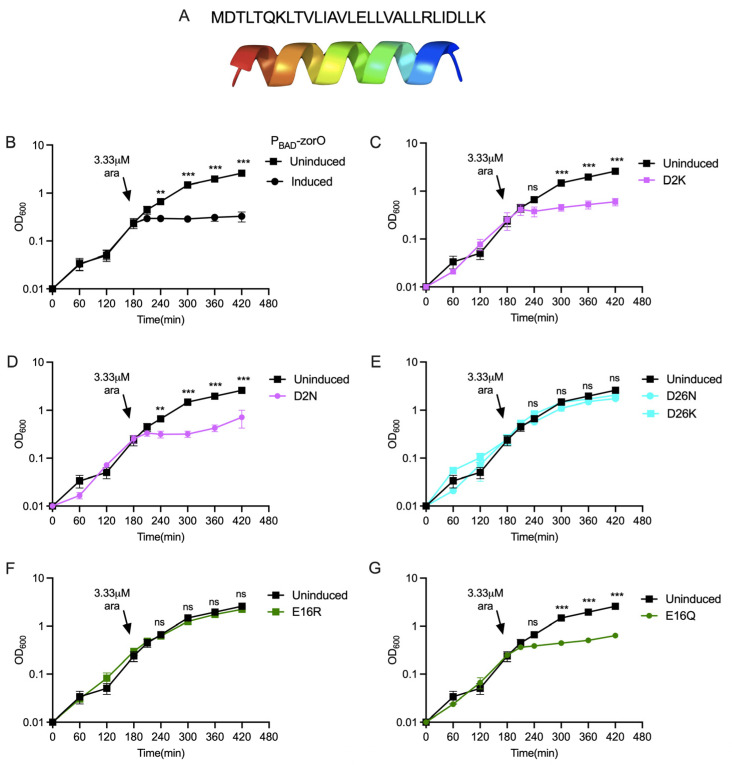
ZorO and the role of negatively charged residues in ZorO mediated toxicity. ZorO sequence and its alpha helical structure predicted by Phyre 2.0 (**A**). Growth curve of *E. coli* UTK007 cells harboring indicated pAZ3 plasmid with ZorO or its variants under an arabinose inducible promoter (P_BAD_). Arrows indicated time of arabinose addition (except for the uninduced control). (**B**) Control; (**C**) aspartate at position 2 converted to lysine (D2K); (**D**) aspartate at position 2 converted to asparagine (D2N); (**E**) aspartate at position 26 converted either to asparagine (D26N) or lysine (D26K); (**F**) glutamate at position 16 converted to arginine (E16R); (**G**) glutamate at position 16 converted to glutamine (E16Q). *n = 3*, shown are mean ± standard deviation. Multiple unpaired t test was performed computing variance for each comparison after arabinose induction. Adjusted *p*-values; ns > 0.05, ** *p* < 0.01, *** *p* < 0.001.

The *zorO* gene encodes for a small protein (29 amino acids in length) that forms a predicted and observed alpha helix (Figure 1A) that localizes to the bacterial inner membrane [19,28,29]. While several computational tools predict ZorO to have a transmembrane domain (Figure S1A), the orientation in the membrane, i.e., the location of the N-terminus or the C-terminus towards the cytoplasm or periplasm, varies between these prediction tools as does the actual length of the transmembrane domain. Regardless, the grand average hydropathy value (GRAVY) is 1.4 for the ZorO sequence indicative of its hydrophobic nature (Figure S1B; [30]). 

ZorO has three positively charged and three negatively charged residues uniformly distributed within its sequence (Figure S1C, red and blue colored residues, respectively). The addition of a FLAG tag (DYKDDDDK) reduces the hydrophobicity index (0.38 for FLAG-ZorO), however, the membrane localization of ZorO was not affected by the FLAG tag [19]. To examine the role of the specific charged residues within the ZorO sequence, we mutated the respective codons to encode either an oppositely charged amino acid or a neutrally charged amino acid (Table S3). The GRAVY index after a single amino acid change was not altered greatly from wild type ZorO except for R23L which was 1.68. 

### 2.2. Specific Charged Amino Acids within the Predicted Transmembrane Domain Are Critical for ZorO Induced Growth Stasis 

For some type I toxins, charged residues are critical for their toxicity and/or formation of larger complexes [23,31,32]. We thus examined the contribution of charged residues in ZorO activity by generating the specific point mutations (Table S3) and monitoring the effects after overexpression. The resulting mutant *zorO* sequences, containing an N-terminus FLAG tag sequence, were overexpressed from the arabinose inducible promoter (P_BAD_) on a high-copy plasmid by adding a final concentration of 3.33 µM (0.00005%) arabinose during mid-log growth phase (OD_600_ nm~0.3) and assessed for growth inhibition. The level of induction (3.33 µM) was chosen as this was the lowest concentration that consistently resulted in cell growth stasis for wild-type ZorO (data not shown). Note, for the purposes herein, if a ZorO variant did not cause growth stasis when induced with 3.33 µM arabinose, it is called a non-toxic variant whereas those variants that induce stasis at this concentration of arabinose are referred to as toxic variants. To maximize ZorO translation, the long 5′ UTR was eliminated, retaining a single-stranded sequence that contained the ribosome binding site (total of 22 nt before the start codon; [27]). 

As previously observed, overexpression of *zorO* results in growth stasis of the cells when compared to uninduced cells (Figure 1B). Mutation of the sequences encoding the negatively charged amino acids, aspartate and glutamate (D2, D26, and E16) had varying effects on ZorO-induced growth stasis. Mutation of the coding sequence for aspartate at position 2 to either lysine (D2K) or asparagine (D2N) maintained ZorO induced toxicity (Figure 1C,D). However, when the sequence for aspartate at position 26 was converted to encode either lysine (D26K) or asparagine (D26N) (Figure 1E), the resulting variants were nontoxic. Conversion of the glutamate at position 16 of ZorO to positively charged arginine (E16R) was nontoxic (Figure 1F) but conversion to charge-neutral glutamine (E16Q) was toxic (Figure 1G).

**Figure 2:**
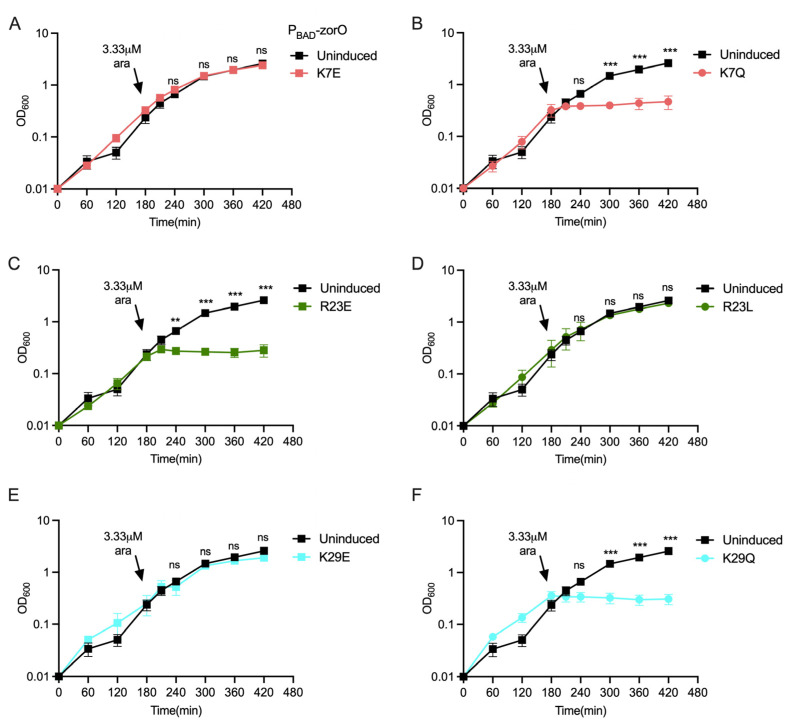
Role of positively charged residues in ZorO mediated toxicity. Growth curve of *E. coli* UTK007 cells harboring indicated pAZ3 plasmid with ZorO or its variants under an arabinose inducible promoter (P_BAD_). Arrows indicated time of arabinose addition (except for the uninduced control). (**A**) lysine at position 7 converted to glutamate (K7E); (**B**) lysine at position 7 converted to glutamine (K7Q); (**C**) arginine at position 23 converted to glutamate (R23E); (**D**) arginine at position 23 converted to leucine (R23L); (**E**) lysine at position 29 converted to glutamate (K29E); (**F**) lysine at position 29 converted to glutamine (K29Q). *n* = 3, shown are mean ± standard deviation. Multiple unpaired t test was performed computing variance for each comparison after arabinose induction. Adjusted *p*-values; ns > 0.05, ** *p* < 0.01, *** *p* < 0.001.

Similar analyses with the positively charged amino acids arginine and lysine (R23, K7, K29) were also performed. Conversion of the lysine residues at the 7th position to glutamate (K7E), arginine at 23rd position to leucine (R23L), and lysine at 29th position to glutamate (K29E) were nontoxic in comparison to overexpression of wild-type *zorO* (Figure 2A,D,E). However, variants for these same codons (generating K7Q, R23E, and K29Q) were toxic (Figure 2B,C,F). Overall, based on these data, altering the charged residues (other than D2) can prevent ZorO mediated growth stasis, yet the effect is specific to the type of mutation and the location of the amino acid within the protein. 

### 2.3. Localization of ZorO after Mutation of the Charged Residues 

Previous overproduction studies indicated that ZorO predominantly localizes to the bacterial inner membrane [19,28], which likely contributes to its effects on membrane polarization, ATP levels and growth inhibition. We wanted to determine if the non-toxic variants of ZorO were due to inappropriate localization and/or reduced expression and/or stability of the ZorO variants. We thus examined the levels of the ZorO variants via western analysis and their localization after induction by 13.3 mM (0.2%) arabinose and separation of the soluble (cytoplasmic) and insoluble (membrane) fractions.

**Figure 3:**
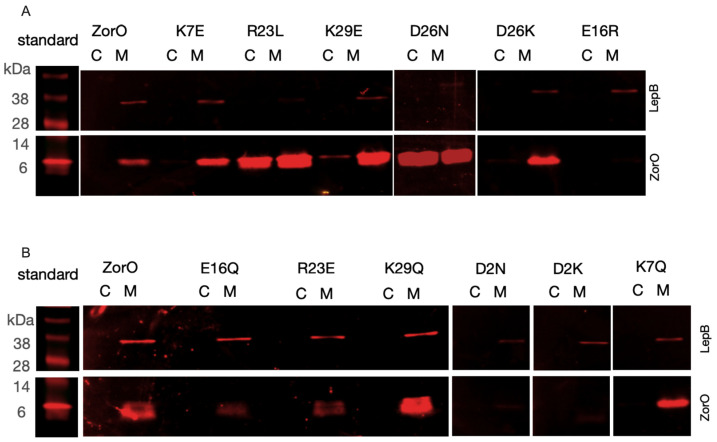
Cellular localization of ZorO mutants. Western blot analysis of the FLAG-tagged ZorO and mutants after subcellular fractionation of the cells induced with 13.3 mM (0.2%) arabinose. Localization of non-toxic ZorO variants (**A**) and toxic variants (**B**). SPA-tagged LepB (membrane protein) was used as control to examine the cellular fractionation. C—cytoplasmic fraction (soluble), M—membrane fraction (insoluble).

Nontoxic ZorO variants did accumulate in the insoluble fraction like wild type ZorO (Figure 3A). Many of these variants also accumulated in the soluble fraction too, except for E16R, though it was not as robustly detected as other variants. Despite these ZorO variants localizing to the membrane, there were no observed effects on growth (Figure 1 and Figure 2) indicating that membrane localization was not sufficient for ZorO mediated cell growth inhibition. The toxic variants of ZorO (D2N, D2K, K7Q, E16Q, R23E and K29Q) were detected primarily in the membrane (insoluble) fraction, similar to wild type ZorO (Figure 3B). 

### 2.4. Impact of Charged Amino Acid Residues on ZorO Induced Membrane Depolarization and ATP Depletion

Apart from growth inhibition, ZorO overproduction results in membrane depolarization and ATP depletion [19,28]. We, therefore, examined how overproduction of the variants impacted membrane polarization and total ATP levels upon induction with 3.33 µM arabinose for 30 min. Membrane depolarization was determined via flow cytometry upon staining with DiBAC4(3) and ATP levels were measured via the BacTiter-Glo reagent and relative luciferase units as described previously [19]. 

**Figure 4:**
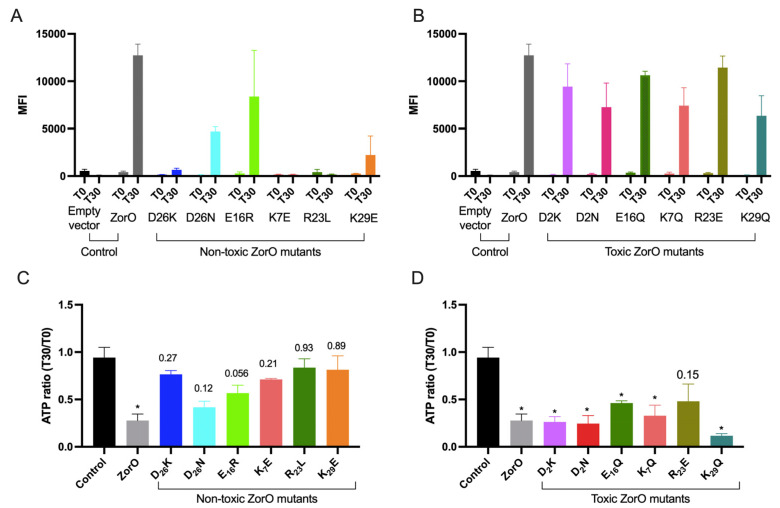
Membrane depolarization and ATP levels upon overproduction of ZorO (WT) and variants under an arabinose inducible promoter (P_BAD_) from a multicopy plasmid in *E. coli* UTK007. Cells were grown as in Figure 1. Mean fluorescence intensity (MFI) as measure of membrane depolarization (**A**,**B**) and ATP ratio (measured as relative fluorescence unit normalized to OD_600_) at 30 min (T30) post arabinose induction compared to T0 (**C**,**D**). *n* = 3, shown are mean ± standard deviation. One-Way ANOVA by comparing the mean of each sample with the mean of an empty vector control was performed with correction for multiple comparison by Dunnett test. Adjusted *p*-values: on the graph, * *p* < 0.05.

Consistent with the results observed for the growth patterns, we found that the membrane depolarization after 30 min (T30) of overexpression of the non-toxic ZorO variants (K7E, R23L, D26K and K29E) were comparable to the basal level before induction (T0) (Figure 4A). Two ZorO variants, D26N and E16R had increased membrane depolarization as compared to the T0 levels. All the ZorO variants that maintained growth inhibition had higher mean fluorescence intensity at 30 min after overexpression indicating membrane depolarization as observed with the wild-type ZorO (Figure 4B). 

We observed a significant drop in cellular ATP levels upon ZorO production as reported for another type I toxin protein TisB [8,19]. To test the impact of the charged residues in ATP levels, we calculated the ratio of ATP levels at 30 min post induction (T30) to that before induction (T0) for cells carrying each ZorO variants and compared that to the cells carrying empty vector (control). The ratio of ATP levels was not statistically significant for the non-toxic ZorO variants (Figure 4C) whereas all the toxic variants except R23E had significant differences in ATP ratio compared to that of the control (Figure 4D). 

### 2.5. Multiple Copies of zorO-orzO Improve Growth in the Presence of Aminoglycosides but Not a Single Copy in the Chromosome

**Figure 5:**
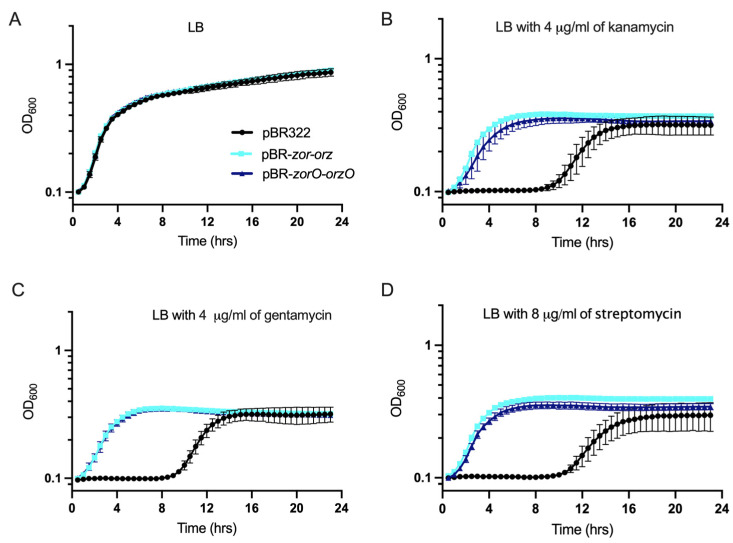
Growth curve of the *E. coli* cells with indicated plasmids (black- empty vector control, turquoise- pBR-*zor-orz*, and blue- pBR-*zorO-orzO*) in the presence of indicated antibiotics. Growth in LB (**A**), LB with 4 µg/mL kanamycin (**B**), 4 µg/mL gentamycin (**C**), and 8 µg/mL streptomycin (**D**) in *E. coli* UTK 007 strain with indicated plasmids. *n* ≥ 3, shown are mean ± standard deviation.

Upon amplifying the *zor-orz* locus by cloning it onto a medium-copy plasmid with its native regulatory elements (pBR322; referred to as pBR-*zor-orz,* contains *zorO-orzO* and *zorP-orzP*), *E. coli* grew better in the presence of kanamycin and gentamycin [19]. To test if the *zorO-orzO* gene pair is sufficient to improve bacterial growth under such conditions, we cloned *zorO-orzO*, with their native regulatory elements, onto pBR322 (pBR-*zorO-orzO*). Cells harboring pBR-*zorO-orzO* grew comparably to cells with pBR-*zor-orz* and emerged from lag phase sooner when compared to the cells with empty vector in the presence of kanamycin, gentamycin, and streptomycin. (Figure 5). These results demonstrate that the *zorO-orzO* pair alone is sufficient to improve growth in the presence of aminoglycosides.

**Figure 6:**
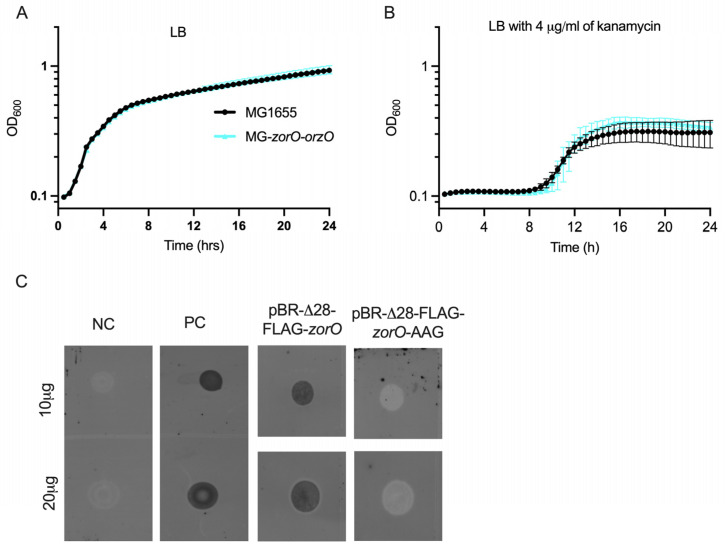
Growth curve of *E. coli* with and without a chromosomal copy of *zorO-orzO* in LB (**A**) and in LB with 4 µg/mL kanamycin (**B**). *n* ≥ 3, shown are mean ± standard deviation. Dot-blot analyses for the detection of ZorO from *E. coli* UTK007 with pBR-Δ28-FLAG-*zorO* (**C**). NC—Negative control with empty vector. PC—Positive control with P_BAD_-FLAG-*zorO*. pBR-Δ28-FLAG-*zorO*-AAG—start codon of *zorO* coding sequence replaced with AAG.

Given our above data, we wanted to determine if kanamycin protection could be induced via production from the chromosome, as opposed to multicopy. We thus integrated *zorO-orzO* into the chromosome of *E. coli* MG1655, referred to as MG-*zorO-orzO*. In LB media, growth of MG-*zorO-orzO* was similar to the parental strain with or without kanamycin (Figure 6A,B). 

### 2.6. ZorO Translation from Its Native Promoter Can Be Detected when the Gene Copy Number Is Increased

As there was no observable improvement in growth in aminoglycosides with only a chromosomal copy of *zorO-orzO*, we wondered if this was due to low translation of the *zorO* mRNA. To date, the translation and localization of ZorO has been demonstrated only via an inducible overexpression system (Figure 3) [19,28]: translation of ZorO from its native regulatory elements has not been shown. The *zorO* transcript possesses a long 5′ UTR that is processed (Δ28) with Δ28-*zorO* translated more robustly than the full-length mRNA [19,27]. Thus, to increase *zorO* translation to aid in its detection, we integrated into the chromosome of *E. coli* MG1655 *zorO* possessing its native promoter elements, truncated UTR (Δ28), and the sequence to encode a FLAG tag (N-terminus, referred to as Δ28-FLAG-*zorO*). However, we were unable to detect translated ZorO using dot-blot or Western blot (data not shown). To conclude if this was a detection limit issue or if translation under native promoter elements does not occur, we cloned Δ28-FLAG-*zorO* into pBR322 (pBR- Δ28-FLAG-*zorO*). While detection via Western blot was variable, we were able to detect ZorO using a dot-blot (Figure 6C), demonstrating for the first-time ZorO translation from its native promoter. 

### 2.7. The Non-Toxic ZorO Mutant R23L Improves Growth in Presence of Kanamycin

**Figure 7:**
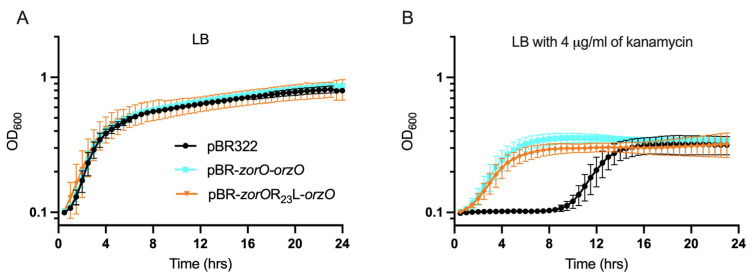
Growth curve of the *E. coli* cells with indicated plasmids (black—empty vector control, turquoise—pBR-*zorO-orzO*, and orange—pBR-*zorO-*R23L*-orzO*) in LB (**A**) and LB with 4 µg/mL kanamycin (**B**). *n* ≥ 3, shown are mean ± standard deviation.

Several type I toxins have been linked to persistence, a mechanism by which a subpopulation of cells can survive lethal dose of antibiotics, mainly by cell growth arrest and reducing the available targets of the antibiotic [10,14,33,34]. In case of the *zor-orz* locus, we observed resistance against aminoglycoside antibiotics, with an increase in MIC [19]. We were curious how the non-toxic mutant ZorO-R23L impacted growth in kanamycin to conclude if the potential beneficial effects of *zorO* could be separated from its inherent toxicity. We performed site directed mutagenesis of pBR-*zorO-orzO* to create pBR-*zorO*-R23L-*orzO*. *E. coli* harboring this plasmid grew similarly to vector control and pBR-*zorO-orzO* in LB media (Figure 7A). Surprisingly, pBR-*zorO*-R23L-*orzO* was also able to improve the growth of *E. coli* in the presence of kanamycin (4 μg/mL) in LB medium and had a shorter lag phase (Figure 7B). This was unexpected especially as the overexpression of R23L does not result in growth inhibition, membrane depolarization or ATP depletion (Figure 2 and Figure 4).

## 3. Discussion

While previous work has examined the translational control and toxicity of ZorO, the role of specific amino acids in ZorO-induced toxicity was not thoroughly examined [19,27,28]. Within, we show that the individual alteration of five out of six charged amino acids (K7, E16, R23, D26, and K29) had impacts on ZorO mediated growth inhibition, membrane depolarization, and ATP depletion concluding the importance of these residues. Importantly though, the effect was dependent on the substituted amino acid. In our previous study, we observed the growth advantage in presence of kanamycin and gentamicin when cells harbored multiple *zor-orz* loci (pBR-*zor-orz*) [19]. Here, we showed that the *zorO-orzO* gene pair is sufficient for this growth advantage in presence of several aminoglycoside antibiotics. Surprisingly, this growth advantage is not dependent upon ZorO mediated effects in the cells (growth stasis, membrane depolarization, and ATP depletion), as the non-toxic ZorO-R23L improved bacterial growth in presence of kanamycin. We also detected, for the first time, FLAG-tagged ZorO via dot-blot under its native promoter when cloned onto the plasmid pBR322. By maintaining native expression elements, amplifying the copies of a type I TA system allows for functional characterization while reducing the complications associated with the inherent toxicity of such a system.

### 3.1. Charged Residues in ZorO Are Important for ZorO Mediated Toxicity

The inner membrane targeting small toxin protein ZorO is hydrophobic and contains six charged amino acid residues (20% of its sequence) distributed within the sequence (Figure 1 and Figure S1). Most prediction tools indicate a transmembrane domain within the sequence, although the length of the predicted domain varies based on the tool used (Figure S1). Single amino acid substitutions or multiple deletion/truncation studies of type I toxins from both Gram-positive and Gram-negative bacteria have shown stretch of hydrophobic residues to be critical [24,35]. In the Fst type I toxin, the charged residues of the C-terminal tail are important for maximal function of the toxin [23]. In ZorO, two charged residues, glutamate at the 16th position and arginine at the 23rd position, are consistently predicted to be in the transmembrane domain regardless of the in silico approach used (Figure S1). Surprisingly, dependent upon the position of the amino acid (16 or 23), alteration to the opposing charge could either maintain toxicity (R23E) or result in a loss of toxicity (E16R) (Figure 1F,G and Figure 2C,D). This could be because of the interaction of ZorO amino acids at specific positions with the membrane and/or with other interacting partners. Mutation of negatively charged aspartate (D2K & D2N) of ZorO was still able to maintain growth inhibition (Figure 1C,D), however, we noted far less accumulation than other variants (Figure 3). None of the predicted tools indicate D2 to be in the transmembrane domain (Figure S1), but this residue may be important for protein stability; we note as well that non-toxic E16R accumulated poorly, which in this case, could explain a possible lack of toxicity. Similar to the effects of ZorO in growth inhibition, the majority of the non-toxic variants had no impacts on membrane depolarization or ATP levels whereas the toxic ZorO variants maintained the membrane depolarization and ATP depletion (Figure 4). The two ZorO variants (D26N and E16R) that did not show growth inhibition at 3.33 µM arabinose induction (Figure 2D,E) resulted in an increase in membrane depolarization (Figure 4A). Both ZorO variants can inhibit growth at higher arabinose concentrations (Figure S2) which may explain the observed membrane depolarization in a subpopulation of the cells. Overall, the positioning of the charged amino acids dictate the toxicity of ZorO; however, more experimentation is needed to understand the exact mechanism for their impacts.

### 3.2. ZorO Mutants Unable to Inhibit Growth Can Accumulate in the Cytoplasm

The membrane localization of the nontoxic ZorO variants revealed that the interaction of ZorO with the inner membrane alone was not sufficient for toxicity (Figure 3). These nontoxic variants of ZorO also accumulated in the cytoplasm: this is because the cells continued to grow after induction with arabinose unlike wildtype ZorO or toxic mutants. Regardless, given the significant accumulation of ZorO in the membrane, membrane localization is not the sole driver for toxicity. Thus, specific interactions, perhaps the formation of stable self-dimers and/or oligomers, or interactions with other membrane proteins are needed to induce toxicity. Further experimentation is ongoing to decipher the exact mechanism of toxicity. 

### 3.3. ZorO Translation under Native Promoter and the Role of zorO-orzO to Improve Bacterial Growth in Presence of Aminoglycoside 

We verified that *zorO-orzO* is sufficient and can improve cellular growth to a similar degree as that of pBR-*zor-orz* against several aminoglycoside antibiotics (Figure 5). As *E. coli* MG1655 lacks a *zorO-orzO* gene pair, we integrated *zorO-orzO* onto the chromosome, however, we did not see improved growth in presence of kanamycin as compared to the wild-type MG1655 strain (Figure 6B). We hypothesized that this could be due to low levels of ZorO production. 

To conclude whether the lack of phenotype with a single copy of *zorO-orzO* in the chromosome was the result of insufficient ZorO levels, we sought to increase *zorO* translation via a strain that lacked two major repressive elements of *zorO*, namely the antitoxin *orzO* and the first 28 nts of *zorO* 5′ UTR. As previously shown, OrzO base pairing to the *zorO* mRNA can result in mRNA cleavage and prevent translation [25,27]. Additionally, the first 28 nts of *zorO* 5′ UTR serve to repress translation: upon processing, a naturally occurring process, the *zorO* mRNA structure is altered and translation is increased [19,27]. We do not yet know the enzymes that process the 5′ UTR, however, the presence of these layers of regulation and possibly other unknown regulators, affects ZorO translation. A similar inhibition of translation of *tisB* toxin is observed: however, integration of a *tisB* variant lacking its antitoxin and inhibitory UTR element allowed for the detection of phenotypes [14]. We thus generated MG-Δ28-FLAG-*zorO* strain that lacks *orzO* and the 28 nt UTR. However, we were not able to detect the translated ZorO protein from the MG-Δ28-FLAG-*zorO* strain via dot-blot even when a large quantity of protein (100 µg) was loaded and there was no improved growth in kanamycin (data not shown). To better conclude the lack of translation is because *zorO* is not translated under cellular conditions or is poorly translated, we then amplified the copy number of Δ28-FLAG-*zorO* via insertion into pBR322. By amplifying the signal, we successfully detected ZorO via dot-blot from cells harboring the pBR-Δ28-FLAG-*zorO* (Figure 6C). 

The lack of phenotype upon chromosomal deletion of a type I TA system is common and therefore demands tools alternative to the ectopic overexpression system. The use of medium-copy plasmid vectors with native regulatory elements (as conducted in this study) is one way to approach this. Further use of more sensitive methods such as mass spectrometry with a focus on small hydrophobic proteins could resolve the issue with the detection of the protein. 

### 3.4. Decoupling ZorO Toxicity and zorO-orzO Mediated Improved Growth in Aminoglycoside

As membrane depolarization has long been associated with aminoglycoside resistance [36,37,38], it was interesting that the non-toxic (R23L) mutant of *zorO* (pBR-*zorO-*R23L-*orzO*) equally improved the growth of the cells in presence of kanamycin (Figure 7B), suggesting that ZorO toxicity can be decoupled from growth in kanamycin. Some random synthetic hydrophobic peptides can confer aminoglycoside resistance by affecting membrane potential and drug uptake after membrane localization [16]. However, those data, combined with ours within, show that membrane depolarization is not essential for the improved growth in kanamycin. While overproduction of ZorO can result in membrane depolarization [19,28], we noted that multiple copies of *zor-orz* do not [19] nor does overproduction of the R23L variant (Figure 4), but these all can induce better growth in the presence of kanamycin (Figure 7B). These results show that protection from kanamycin is via an alternative mechanism independent of membrane depolarization. This opens door to more questions. One, could the small hydrophobic protein ZorO have a dual function? The first function would be as a toxin (growth inhibition, membrane depolarization, ATP depletion) and the second, improving growth in presence of aminoglycoside. This could explain the results observed with ZorO-R23L mutant. We note that frameshift mutations within the *ibsC* toxin are still toxic, so what is needed for “toxicity” can be highly variable [39], thus it is possible that the substitution mutation of R23L in ZorO preserves its protective, second function. Two, is the function of ZorO dependent on its concentration? We see the toxic effects of ZorO only upon ectopic overproduction from an inducible promoter whereas we have seen the beneficial growth in presence of aminoglycoside when controlled by its native regulatory elements. Thus, there could be some concentration gradient determining the function and/or effects of ZorO. When ZorO levels are appropriate, it interacts with its true target whereas artificial overproduction results in saturation and possible interactions with non-native targets. Three, is the *zorO-orzO* mediated improved growth in kanamycin a function of the interaction between *zorO* and OrzO? If this is the case, ZorO being toxic (WT) or non-toxic (R23L) would not affect the role of *zorO-orzO* in improved growth in kanamycin as the region of base pairing is maintained in pBR-*zor-orzO* and pBR-*zorO*-R23L-*orzO*. Finally, what is the contribution of RNA stability? Perhaps the increased levels of a stable mRNA impact ribosomal activity, resulting in aminoglycoside protection. These findings, in combination with a new study suggesting that membrane depolarization is a downstream consequence of kanamycin treatment [40], supports the need for additional research to determine the mechanism behind *zorO-orzO*-mediated aminoglycoside protection. 

## 4. Conclusions

Overall, the results from our study shed light on the importance of charged amino acids in the toxicity (growth inhibition, membrane depolarization, ATP depletion) mediated by the type I toxin protein ZorO. How charged amino acids contribute to toxicity though is not simple and their impacts vary depending on the position of the residue and how the amino acid is altered. The results of these alterations most likely affect its interaction with either itself to form a dimer/oligomer, or with membrane components or with other partner molecules within the cell. The effect of ZorO and the role of *zorO-orzO* in improved growth in presence of kanamycin is independent of toxicity as the non-toxic mutant *zorO*-R23L-*orzO* was also able to improve the growth of the cells in presence of kanamycin: this adds further questions regarding the possible biological functions of type I toxin-antitoxin systems. Further, the results from our study demonstrate that type I toxin proteins can be detected from their native promoter via amplification of its copy number without the complication of its inherent toxicity.

## 5. Materials and Methods

### 5.1. Bacterial Strains and Plasmids 

All bacterial strains and plasmids used in this study are listed in Table S1. The sequences of all oligonucleotides are listed in Table S2.

### 5.2. Growth Conditions

*Overproduction studies.* The overproduction of ZorO variants was described previously [25]. Briefly, *E. coli* UTK007, a derivative of MG1655 with constitutive *araE*, was transformed fresh (not older than 7 days) with the indicated plasmid derivatives of pAZ3 using electroporation. The resulting transformants were grown overnight in 5 mL of LB medium (with 25 μg/mL chloramphenicol final concentration) at 37 °C with shaking and diluted to an OD_600_ of 0.01. At an OD_600_ of ~0.3, arabinose was added to final concentrations of 13.32 mM (0.2%) or 3.33 μM (0.00005%) as indicated in the text. *E. coli* UTK007 carrying the empty vector (pAZ3) or uninduced (no arabinose) were used as controls. OD_600_ was measured and averages ± standard deviations for a minimum of three replicates are shown.

*Microplate growth curves.* Overnight cultures were grown in LB medium. Ampicillin (100 μg/mL final concentration) was added when testing cells transformed with pBR322. Overnight cultures were diluted to an OD_600_ of 0.2 in 1 mL sterile phosphate-buffered saline. A 96-well microplate was prepared by adding 190 μL of the culture media (LB) containing the antibiotics as indicated. Then, 10 μL of 0.2 OD_600_ culture (diluted in PBS from overnight culture) was added to obtain a 200 μL total volume of 0.01 OD_600_ culture. Absorbance was recorded on a Gen5^TM^ Microplate reader (BioTek Instruments, Inc., Winooski, VT, USA) every 30 min, after 15 s of shaking, for 24 h at 37 °C. Shown are averages ± standard deviations for a minimum of three replicates. 

### 5.3. Plasmid and Strain Construction

Specific point mutants were created by site-directed mutagenesis of the pAZ3-FLAG-*zorO* plasmid as previously described [25] using the indicated oligonucleotides in Table S2. Briefly, plasmids amplified with indicated oligonucleotides were DpnI digested, purified, and transformed into *E. coli* TOP10 cells. Plasmid DNA was extracted and confirmed via sequencing.

The pBR322 derived plasmids were constructed by using Gibson assembly. The inserts were amplified using the indicated oligonucleotides in Table S2. The insert for Δ28-FLAG-*zorO* was amplified from EDL933- Δ28-FLAG-*zorO* [19].

To construct MG-*zorO-orzO, zorO-orzO* was linked to a chloramphenicol cassette (flanked with FRT sites) via Gibson assembly on a pBR plasmid using oligonucleotides EF1856, EF1857, EF1858, EF1859 (Table S2). The *zorO-orzO*-FRT cm was then recombineered into NM1100 strain after amplifying with the oligonucleotides EF1864A and EF1865A (Table S2), followed by P1 transduction into the MG1655 *E. coli*. The chloramphenicol cassette was then removed via pCP20 treatment [41].

### 5.4. Membrane Depolarization Assay

Membrane depolarization assays were performed as described previously [19], using the membrane potential sensitive dye Bis-(1,3-dibtylbarbituric acid) trimethine oxonol (DiBAC_4_-3; Invitrogen TM). A 25 mg/mL stock solution was prepared in dimethyl sulfoxide. At indicated time points, 50–100 μL of culture was removed, flooded with 4 mL 1× phosphate-buffered saline (PBS) and centrifuged for 10 min at 4 °C. Cells were resuspended in 1 mL PBS and stained with DiBAC_4_-3 (10 μg/mL final concentration) for 20 min in the dark. After washing with 1× PBS twice, cells were re-suspended in 0.5 mL of 1× PBS and analyzed by flow cytometry in an LSR II flow cytometer (Becton Dickinson Company, Franklin Lakes, NJ, USA) with a 488 nm laser. Samples were run at ~3000 events per second and fluorescence was collected in the fluorescein isothiocyanate channel. Data were analyzed using the FlowJo v10.7 software package (FlowJo LLC, Ashland, OR, USA) such that the cells showing at least 10^3^ abu or more fluorescence were gated and mean fluorescence intensity (MFI) was obtained. All tests were carried out with a minimum of three biological replicates. Shown are averages ± standard deviations for a minimum of three replicates.

### 5.5. ATP Measurements

ATP levels were measured as described previously [19], using the BacTiter Glo ATP Assay System Bioluminescence Detection Kit for ATP Measurement (Promega Corporation, Madison, WI, USA) according to the manufacturer’s instructions. The observed relative luciferase unit (RLU) values were normalized to OD_600_ at the time of cell harvest. A minimum of three biological replicates were performed per strain/condition. Shown are averages ± standard deviations for a minimum of three replicates.

### 5.6. Western Blot and Dot-Blot Analyses

Western blot and dot-blot analyses were performed as described previously [19]. Cells (50 mL) were harvested either 30 min after overexpression for pAZ3-derived plasmids or at an OD_600_ ~0.3 for other cells and then subjected to via bead beating. Separation of the insoluble fraction (membrane) from the soluble fraction was performed via ultracentrifugation (100,000× *g*, 4 °C, 45 min) to yield a supernatant (cytoplasmic proteins) and a pellet (membrane proteins) [42]. The pellet was washed with cold 1× PBS and resuspended in 100 μL 1× PBS. Protein concentration was measured using a Bradford Protein Assay. For western analyses, protein samples (10 μg) were separated on an SDS-PAGE gel, transferred to an immobilon-FL membrane, and probed with a rat derived α-FLAG tag primary antibody (BioLegend) and an α-IgG secondary antibody (LI-COR Biosciences) that fluoresces at 680 nm. LepB was tagged with a SPA tag (UTK102) to serve as a loading control [19,42]. 

Dot-blots were performed by spot inoculating a nitrocellulose membrane with 5 μL of the sample containing 10 μg or 20 μg of protein and processing the membrane as above (probed with α-FLAG primary antibody α-IgG secondary antibody) [43].

## Data Availability

All data are available within the text.

## Supplementary Materials

The following supporting information can be downloaded at: https://www.mdpi.com/article/10.3390/toxins15010032/s1, Table S1: Bacterial strains and plasmids used in this study, Table S2: Oligonucleotides used in this study, Table S3: Point mutants of the charged amino acid residues of ZorO and their respective GRAVY index, Figure S1: Structure and hydropathy prediction of ZorO, Figure S2: Growth curve of *E. coli* UTK007 cells carrying indicated pAZ3 plasmid vector.

## References

[1] Brantl S. (2012). Bacterial type I toxin-antitoxin systems. RNA Biol..

[2] Wen J., Fozo E.M. (2014). sRNA antitoxins: More than one way to repress a toxin. Toxins.

[3] Nonin-Lecomte S., Fermon L., Felden B., Pinel-Marie M.-L. (2021). Bacterial type I toxins: Folding and membrane interactions. Toxins.

[4] Guo Y., Quiroga C., Chen Q., McAnulty M.J., Benedik M.J., Wood T.K., Wang X. (2014). RalR (a DNase) and RalA (a small RNA) form a type I toxin-antitoxin system in *Escherichia coli*. Nucleic Acids Res..

[5] Kawano M., Aravind L., Storz G. (2007). An antisense RNA controls synthesis of an SOS-induced toxin evolved from an antitoxin. Mol. Microbiol..

[6] Wilmaerts D., Bayoumi M., Dewachter L., Knapen W., Mika J.T., Hofkens J., Dedecker P., Maglia G., Verstraeten N., Michiels J. (2018). The persistence-inducing toxin HokB forms dynamic pores that cause ATP leakage. MBio.

[7] Schneider V., Wadhwani P., Reichert J., Bürck J., Elstner M., Ulrich A.S., Kubař T. (2019). Tetrameric Charge-Zipper Assembly of the TisB peptide in membranes-computer simulation and experiment. J. Phys. Chem. B.

[8] Unoson C., Wagner E.G.H. (2008). A small SOS-induced toxin is targeted against the inner membrane in *Escherichia coli*. Mol. Microbiol..

[9] Wilmaerts D., Dewachter L., De Loose P.-J., Bollen C., Verstraeten N., Michiels J. (2019). Hokb monomerization and membrane repolarization control persister awakening. Mol. Cell.

[10] Verstraeten N., Knapen W.J., Kint C.I., Liebens V., Van den Bergh B., Dewachter L., Michiels J.E., Fu Q., David C.C., Fierro A.C. (2015). Obg and membrane depolarization are part of a microbial bet-hedging strategy that leads to antibiotic tolerance. Mol. Cell.

[11] Gurnev P.A., Ortenberg R., Dörr T., Lewis K., Bezrukov S.M. (2012). Persister-promoting bacterial toxin TisB produces anion-selective pores in planar lipid bilayers. FEBS Lett..

[12] Wagner E.G.H., Unoson C. (2012). The toxin-antitoxin system *tisB-istR1*: Expression, regulation, and biological role in persister phenotypes. RNA Biol..

[13] Dörr T., Vulić M., Lewis K. (2010). Ciprofloxacin causes persister formation by inducing the TisB toxin in *Escherichia coli*. PLoS Biol..

[14] Berghoff B.A., Hoekzema M., Aulbach L., Wagner E.G.H. (2017). Two regulatory RNA elements affect TisB-dependent depolarization and persister formation. Mol. Microbiol..

[15] Vogel J., Argaman L., Wagner E.G.H., Altuvia S. (2004). The small RNA IstR inhibits synthesis of an SOS-induced toxic peptide. Curr. Biol..

[16] Knopp M., Gudmundsdottir J.S., Nilsson T., König F., Warsi O., Rajer F., Ädelroth P., Andersson D.I. (2019). De novo emergence of peptides that confer antibiotic resistance. MBio.

[17] Kawano M., Oshima T., Kasai H., Mori H. (2002). Molecular characterization of long direct repeat (LDR) sequences expressing a stable mRNA encoding for a 35-amino-acid cell-killing peptide and a cis-encoded small antisense RNA in *Escherichia coli*. Mol. Microbiol..

[18] Fozo E.M., Kawano M., Fontaine F., Kaya Y., Mendieta K.S., Jones K.L., Ocampo A., Rudd K.E., Storz G. (2008). Repression of small toxic protein synthesis by the Sib and OhsC small RNAs. Mol. Microbiol..

[19] Bogati B., Wadsworth N., Barrera F., Fozo E.M. (2021). Improved growth of *Escherichia coli* in aminoglycoside antibiotics by the *zor-orz* toxin-antitoxin system. J. Bacteriol..

[20] Patel S., Weaver K.E. (2006). Addiction toxin Fst has unique effects on chromosome segregation and cell division in *Enterococcus faecalis* and *Bacillus subtilis*. J. Bacteriol..

[21] Göbl C., Kosol S., Stockner T., Rückert H.M., Zangger K. (2010). Solution structure and membrane binding of the toxin *fst* of the *par* addiction module. Biochemistry.

[22] Jahn N., Brantl S., Strahl H. (2015). Against the mainstream: The membrane-associated type I toxin BsrG from *Bacillus subtilis* interferes with cell envelope biosynthesis without increasing membrane permeability. Mol. Microbiol..

[23] Holmes A., Sadlon J., Weaver K. (2021). Charged residues flanking the transmembrane domain of two related toxin-antitoxin system toxins affect host response. Toxins.

[24] Mok W.W.K., Patel N.H., Li Y. (2010). Decoding toxicity: Deducing the sequence requirements of IbsC, a type I toxin in *Escherichia coli*. J. Biol. Chem..

[25] Wen J., Won D., Fozo E.M. (2014). The ZorO-OrzO type I toxin-antitoxin locus: Repression by the OrzO antitoxin. Nucleic Acids Res..

[26] Fozo E.M., Makarova K.S., Shabalina S.A., Yutin N., Koonin E.V., Storz G. (2010). Abundance of type I toxin-antitoxin systems in bacteria: Searches for new candidates and discovery of novel families. Nucleic Acids Res..

[27] Wen J., Harp J.R., Fozo E.M. (2017). The 5΄ UTR of the type I toxin ZorO can both inhibit and enhance translation. Nucleic Acids Res..

[28] Otsuka Y., Ishikawa T., Takahashi C., Masuda M. (2019). A short peptide derived from the ZorO toxin functions as an effective antimicrobial. Toxins.

[29] Kelley L.A., Mezulis S., Yates C.M., Wass M.N., Sternberg M.J.E. (2015). The Phyre2 web portal for protein modeling, prediction and analysis. Nat. Protoc..

[30] Kyte J., Doolittle R.F. (1982). A simple method for displaying the hydropathic character of a protein. J. Mol. Biol..

[31] Wilmaerts D., De Loose P.-J., Vercauteren S., De Smedt S., Verstraeten N., Michiels J. (2021). Functional analysis of cysteine residues of the Hok/Gef type I toxins in *Escherichia coli*. FEMS Microbiol. Lett..

[32] Steinbrecher T., Prock S., Reichert J., Wadhwani P., Zimpfer B., Bürck J., Berditsch M., Elstner M., Ulrich A.S. (2012). Peptide-lipid interactions of the stress-response peptide TisB that induces bacterial persistence. Biophys. J..

[33] Page R., Peti W. (2016). Toxin-antitoxin systems in bacterial growth arrest and persistence. Nat. Chem. Biol..

[34] Cheverton A.M., Gollan B., Przydacz M., Wong C.T., Mylona A., Hare S.A., Helaine S. (2016). A *Salmonella* toxin promotes persister formation through acetylation of tRNA. Mol. Cell.

[35] Weaver K.E., Reddy S.G., Brinkman C.L., Patel S., Bayles K.W., Endres J.L. (2009). Identification and characterization of a family of toxin-antitoxin systems related to the *Enterococcus faecalis* plasmid pAD1 par addiction module. Microbiology.

[36] Schwarz S., Loeffler A., Kadlec K. (2017). Bacterial resistance to antimicrobial agents and its impact on veterinary and human medicine. Vet. Dermatol..

[37] Krause K.M., Serio A.W., Kane T.R., Connolly L.E. (2016). Aminoglycosides: An overview. Cold Spring Harb. Perspect. Med..

[38] Wilson D.N. (2014). Ribosome-targeting antibiotics and mechanisms of bacterial resistance. Nat. Rev. Microbiol..

[39] Huang X., Chen R., Sun M., Peng Y., Pu Q., Yuan Y., Chen G., Dong J., Du F., Cui X. (2020). Frame-shifted proteins of a given gene retain the same function. Nucleic Acids Res..

[40] Bruni G.N., Kralj J.M. (2020). Membrane voltage dysregulation driven by metabolic dysfunction underlies bactericidal activity of aminoglycosides. Elife.

[41] Doublet B., Douard G., Targant H., Meunier D., Madec J.-Y., Cloeckaert A. (2008). Antibiotic marker modifications of lambda Red and FLP helper plasmids, pKD46 and pCP20, for inactivation of chromosomal genes using PCR products in multidrug-resistant strains. J. Microbiol. Methods.

[42] Fontaine F., Fuchs R.T., Storz G. (2011). Membrane localization of small proteins in *Escherichia coli*. J. Biol. Chem..

[43] Hemm M.R., Paul B.J., Miranda-Ríos J., Zhang A., Soltanzad N., Storz G. (2010). Small stress response proteins in *Escherichia coli*: Proteins missed by classical proteomic studies. J. Bacteriol..

