# Supplementary material for: Charged Amino Acids Contribute to ZorO Toxicity
Source: Toxins (Basel). 2022 Dec 31;15(1):32. doi: 10.3390/toxins15010032 (PMC9860968; doi:10.3390/toxins15010032)
Supplement: Supplementary file 1 [file toxins-15-00032-s001.zip › toxins-2127780-supplementary.pdf]

Table S1. Bacterial strains and plasmids used in this study.

Strain or plasmid	Relevant characteristics	Source	Primers
<u>Bacterial strains</u>			
E. coli MG1655	*E. coli* F^{-} $^{-}$ *ilvG*$^{-}$ *rfb*-50 *rph*-1	Lab stock	
E. coli Top10	Cloning vector	Lab stock	
UTK007	DJ480 PCP-*araE*	[1]	
UTK102	UTK007-*lepB*-SPA	[2]	
MG- Δ28-FLAG-*zorO*	MG1655 recombineered with P_{zorO}-Δ28UTR-FLAG*zorO*	[2]	
MG-*zorO*-*orzO*	MG1655 recombineered with *zorO*-*orzO*	This study	EF1864A, EF1865A
<u>Plasmids</u> pAZ3	Cm(R), P_{BAD} promoter	[3]	
pAZ3-FLAG-*zorO*	Cm(R)	[2]	
pAZ3-*zorO*-D2N	Cm(R)	This study	EF1576 EF1577
pAZ3- *zorO*-D2K	Cm(R)	This study	EF1574, EF 1575
pAZ3- *zorO*-K7Q	Cm(R)	This study	EF1580, EF1581
pAZ3-*zorO*-K7E	Cm(R)	This study	EF1578, EF1579
pAZ3-*zorO*-E16Q	Cm(R)	This study	EF1184, EF1185
pAZ3-*zorO*-E16R	Cm(R)	This study	EF1215, EF1216
pAZ3-*zorO*-R23L	Cm(R)	This study	EF1186, EF1187
pAZ3-*zorO*-R23E	Cm(R)	This study	EF1217, EF1218

Table S1. Continued.

Strain or plasmid	Relevant characteristics	Source	Primers
pAZ3-*zorO*-D26N	Cm(R)	This study	EF1311, EF1312
pAZ3- *zorO*-D26K	Cm(R)	This study	EF1309, EF1310
pAZ3- *zorO*-K29Q	Cm(R)	This study	EF1315, EF1316
pAZ3-*zorO*-K29E	Cm(R)	This study	EF1313, EF1314
pBR322	Amp(R), Tet(R)	[4]	
pBR-*zor*-*orz*	Amp(R)	[2]	
pBR-*zorO*-*orzO*	Amp(R)	This study	EF1835, EF1836, EF1837, EF1838
pBR-*zorO*-R23L*orzO*	Amp(R)	This study	EF1186, EF1187
pBR-plac-*orzO*	Amp(R)	[1]	
pBR-*zorO*	Amp(R)	[2]	
pBR-Δ28-FLAG*zorO*	Amp(R)	This study	EF1836, EF1839, EF1837, EF1840

Table S2. Oligonucleotides used in this study.

Name	Oligonucleotide sequence, 5′→3′
EF1184	GCTCATTGCCGTACTGCAGTTATTAGTGGCTCTG
EF1185	CAGAGCCACTAATAACTGCAGTACGGCAATGAGC
EF1186	GTTATTAGTGGCTCTGTTACTGTTGATTGATTTGTTGAAG
EF1187	CTTCAACAAATCAATCAACAGTAACAGAGCCACTAATAAC
EF1215	CGTGCTCATTGCCGTACTGCGGTTATTAGTGGCTCTGTTACG
EF1216	CGTAACAGAGCCACTAATAACCGCAGTACGGCAATGAGCACG
EF1217	GAGTTATTAGTGGCTCTGTTAGAGTTGATTGATTTGTTGAAGTAAC
EF1218	GTTACTTCAACAAATCAATCAACTCTAACAGAGCCACTAATAACTC
EF1309	GTGGCTCTGTTACGGTTGATTAAGTTGTTGAAGTAACGGGCAAATTG
EF1310	CAATTTGCCCGTTACTTCAACAACTTAATCAACCGTAACAGAGCCAC
EF1311	GTGGCTCTGTTACGGTTGATTAATTTGTTGAAGTAACGGGCAAATTG
EF1312	CAATTTGCCCGTTACTTCAACAAATTAATCAACCGTAACAGAGCCAC
EF1313	CTGTTACGGTTGATTGATTTGTTGGAGTAACGGGCAAATTGAAACGCATC
EF1314	GATGCGTTTCAATTTGCCCGTTACTCCAACAAATCAATCAACCGTAACAG
EF1315	CTGTTACGGTTGATTGATTTGTTGCAGTAACGGGCAAATTGAAACGCATC
EF1316	GATGCGTTTCAATTTGCCCGTTACTGCAACAAATCAATCAACCGTAACAG
EF1574	GATTATAAAGATGATGATGATAAAAAGACGCTGACACAAAAGTTAACCGTG
EF1575	CACGGTTAACTTTTGTGTCAGCGTCTTTTTATCATCATCATCTTTATAATC
EF1576	GATTATAAAGATGATGATGATAAAAACACGCTGACACAAAAGTTAACCGTG
EF1577	CACGGTTAACTTTTGTGTCAGCGTGTTTTTATCATCATCATCTTTATAATC
EF1578	GATGATAAAGACACGCTGACACAAGAGTTAACCGTGCTCATTGCCGTACTG
EF1579	CAGTACGGCAATGAGCACGGTTAACTCTTGTGTCAGCGTGTCTTTATCATC
EF1580	GATGATAAAGACACGCTGACACAACAGTTAACCGTGCTCATTGCCGTACTG

Table S2. (continued).

Name	Oligonucleotide sequence, 5′→3′
EF1581	CAGTACGGCAATGAGCACGGTTAACTGTTGTGTCAGCGTGTCTTTATCATC
EF1835	CATCGTCATCCTCGGCACCGTCCATCGATTTTATATTCAACGC
EF1836	GTCCCTGATGGTCGTCATCTACGGATCCCCCCTTAGATGCG
EF1837	CGCATCTAAGGGGGGATCCGTAGATGACGACCATCAGGGAC
EF1838	GCGTTGAATATAAAATCGATGGACGGTGCCGAGGATGACGATG
EF1839	GCGCTCATCGTCATCCTCGGCACCGTCCGAATAAATTATAACCATATCC
EF1840	GGATATGGTTATAATTTATTCGGACGGTGCCGAGGATGACGATGAGCGC
EF1856	GTAGATGACGACCATCAGGGACCATATGAATATCCTCCTTAG
EF1857	CGAGCGATCCTTGAAGCTGTGTAGGCTGGAGCTGCTTCG
EF1858	CGAAGCAGCTCCAGCCTACACAGCTTCAAGGATCGCTCG
EF1859	CTAAGGAGGATATTCATATGGTCCCTGATGGTCGTCATCTAC
EF1864A	CTCTAAATAATTCAAATTGGTCCGATCCGGCGCAACGTCCCCATCGATTTTAT ATTCAACGCACC
EF1865A	CATCCCTGAAATGTATTAATGAAATAAACGATATTTTACGCGCGTGTAGGCT GGAGCTGCTTCG
EF1869B	CTCTAAATAATTCAAATTGGTCCGATCCGGCGCAACGTCCCCATCGTCATCCT CGGCACCGGATCC

Table S3. Point mutants of the charged amino acid residues of ZorO and their respective GRAVY index.

Amino acid	Position	Mutated to	Symbol	GRAVY index [5]
	Wild type		ZorO	1.40
Aspartate	2	Asparagine	D2N	1.39
		Lysine	D2K	1.38
Lysine	7	Glutamine	K7Q	1.41
		Glutamate	K7E	1.40
Glutamate	16	Arginine	E16R	1.36
		Glutamine	E16Q	1.40
Arginine	23	Leucine	R23L	1.68
		Glutamate	R23E	1.43
Aspartate	26	Asparagine	D26N	1.40
		Lysine	D26K	1.38
Lysine	29	Glutamine	K29Q	1.41
		Glutamate	K29E	1.41

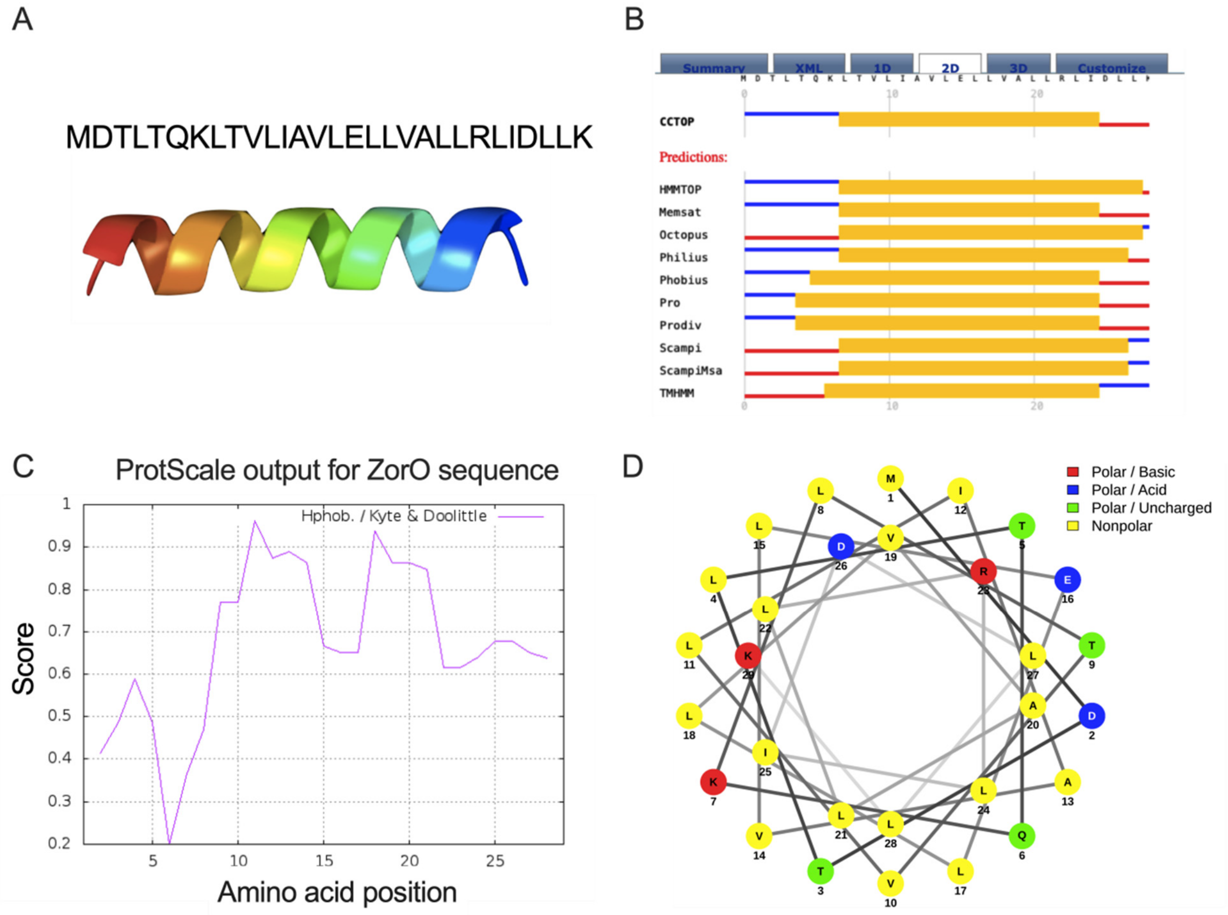

Figure S1. Structure and hydropathy prediction of ZorO. Alpha-helical structure of ZorO predicted by Phyre 2.0 (A). Predicted transmembrane domain and orientation (B); Blue line at N-terminus indicates N-terminus towards the periplasm, yellow bar represents the predicted transmembrane domain. The ProtScale value based on hydrophobicity scale of ZorO sequence (C). Helical wheel projection of ZorO (D).

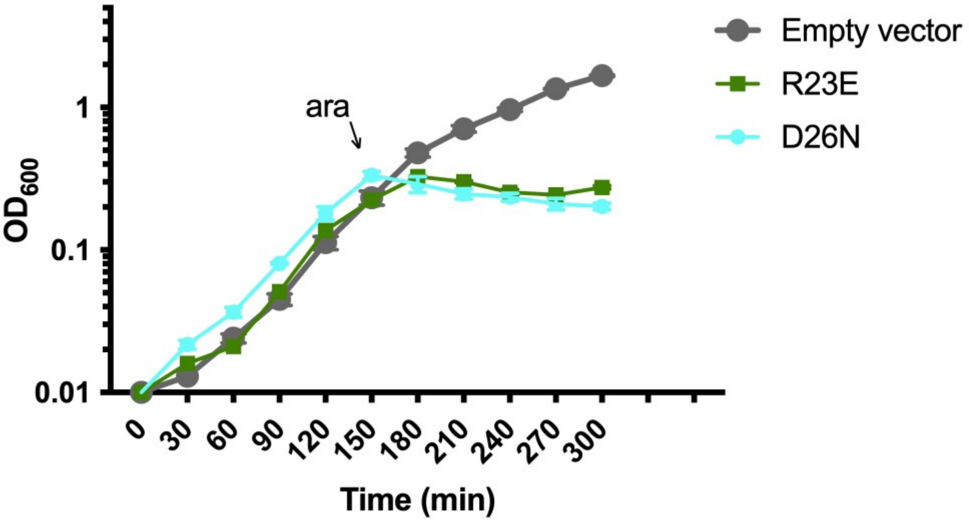

Figure S2. Growth curve of *E. coli* UTK007 cells carrying indicated pAZ3 plasmid vector. Cells were grown in LB medium until mid-log phase and induced by arabinose (6.66 M for R23E and 66.6 M for D26N) *n=3*, shown are mean ± standard deviation. .

References

1. Wen, J.; Won, D.; Fozo, E. M. The ZorO-OrzO type I toxin-antitoxin locus: repression by the OrzO antitoxin. *Nucleic Acids Res.* **2014**, *42*, 1930–1946, doi:10.1093/nar/gkt1018.
2. Bogati, B.; Wadsworth, N.; Barrera, F.; Fozo, E. M. Improved growth of *Escherichia coli* in aminoglycoside antibiotics by the *zor-orz* toxin-antitoxin system. *J. Bacteriol.* **2021**, JB0040721, doi:10.1128/JB.00407-21.
3. Kawano, M.; Aravind, L.; Storz, G. An antisense RNA controls synthesis of an SOS-induced toxin evolved from an antitoxin. *Mol. Microbiol.* **2007**, *64*, 738–754, doi:10.1111/j.13652958.2007.05688.x.
4. Bolivar, F.; Rodriguez, R. L.; Greene, P. J.; Betlach, M. C.; Heyneker, H. L.; Boyer, H. W.; Crosa, J. H.; Falkow, S. Construction and characterization of new cloning vehicles. II. A multipurpose cloning system. *Gene* **1977**, *2*, 95–113, doi:10.1016/0378-1119(77)90000-2.
5. Kyte, J.; Doolittle, R. F. A simple method for displaying the hydropathic character of a protein. *J. Mol. Biol.* **1982**, *157*, 105–132, doi:10.1016/0022-2836(82)90515-0.
